# Branched endovascular aneurysm repair complicated by disseminated intravascular coagulation while using tocilizumab

**DOI:** 10.1016/j.jvscit.2025.101936

**Published:** 2025-08-14

**Authors:** Ella Lubberdink, Saskia E.M. Schols, Teba Alnima, Mark Dirven

**Affiliations:** a Department of Surgery, Radboud University Medical Center, Nijmegen, the Netherlands; b Department of Hematology, Radboud University Medical Center, Nijmegen, the Netherlands; c Department of Internal Medicine, Vascular Medicine, Radboud University Medical Center, Nijmegen, the Netherlands; d Department of Vascular Surgery, Radboud University Medical Center, Nijmegen, the Netherlands

**Keywords:** Branched endovascular aneurysm repair, Crawford type III thoraco-abdominal aneurysm, Disseminated intravascular coagulation, Tocilizumab

## Abstract

Disseminated intravascular coagulation (DIC) has been reported as a complication following endovascular procedures. We present the case of a 69-year-old female with giant cell arteritis on tocilizumab therapy, who underwent a first-stage branched endovascular aneurysm repair for a Crawford type III thoracoabdominal aneurysm. Postoperatively, she developed DIC. After excluding other causes, we assumed endoleakage through the celiac branch combined with tocilizumab-induced hypofibrinogenemia as the primary cause. The second stage was expedited and tocilizumab discontinued. The DIC resolved within weeks. Although rare, hemostatic disbalances may occur after endovascular procedures. Tocilizumab might have facilitated the progression of DIC under these circumstances.

Disseminated intravascular coagulation (DIC) is characterized by persistent activation of the coagulation system, leading to excessive thrombin and fibrin production. This results in systemic consumption of platelets and coagulation factors, increasing the risk of thrombosis and bleeding. DIC can be life-threatening, often progressing to multiorgan failure. It is secondary to an underlying condition, with sepsis and malignancy being the most common causes.^1^ Singh et al cited mortality rates ranging from 45% to 78% reported in previous studies.^2^ Effective management requires addressing the primary condition responsible for this phenomenon.

We present the case of a 69-year-old female with giant cell arteritis (GCA) on tocilizumab therapy, who developed DIC after a first-stage branched endovascular aneurysm repair (BEVAR), which resolved after the completion of the BEVAR and discontinuation of tocilizumab. Informed consent was obtained from the patient for publication.

## Case report

A 69-year-old female patient was admitted to Radboud University Medical Center for treatment of a 65-mm asymptomatic Crawford type III thoracoabdominal aneurysm. Her medical history included a Coxiella Burnetti infection, radiculopathy of L5, mild obstructive sleep apnea, hypertension, polymyalgia rheumatica, GCA, and a supracoronary ascending aorta replacement. For GCA, she started using tocilizumab in 2018, remaining stable on 8 mg/kg every 4 weeks, combined with 2.5 mg prednisone. During the ascending aortic repair in 2016, the patient was receiving methotrexate instead of tocilizumab. Other medications consisted of amlodipine, atenolol, calcium carbonate, clopidogrel, cholecalciferol, liraglutide, pantoprazole, paracetamol, paroxetine, Plantago ovata granulate, prednisone, and rosuvastatin.

Due to growth of her thoracoabdominal aneurysm and subsequent rupture risk, a two-staged BEVAR was offered, as open repair was less suitable due to morbid obesity (body mass index, 36.8 kg/m^2^) and extensive comorbidity. The first stage involved placement of a BEVAR with branches to the renal arteries and mesenteric artery, leaving the celiac trunk branch open. The second stage involved completing the celiac branch.

**Fig 1:**
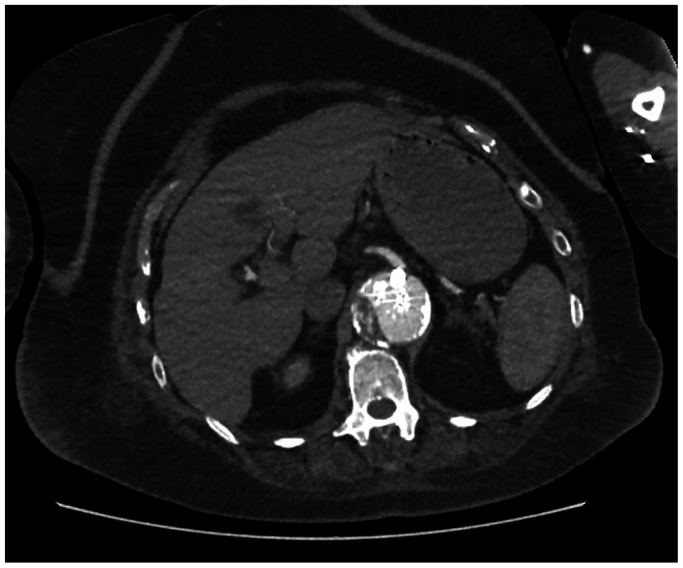
Computed tomography angiography performed after the first-stage branched endovascular aneurysm repair (BEVAR), showing a type III endoleak caused by intentional preservation of the celiac trunk branch.

In August 2024, the first stage was performed with percutaneous access via both common femoral arteries and eventually the right brachial artery. Heparin (5000 IE) was administered. BeGraft plus stents (Bentley InnoMed) were placed, and the aneurysm remained in circulation with the truncus branch deliberately left open, causing a type III endoleak (Fig 1).

**Fig 2:**
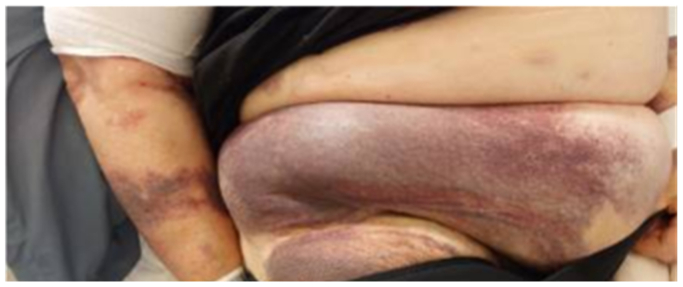
Large hematoma originating from the right groin, with additional large hematomas present in other areas of the body.

**Fig 3:**
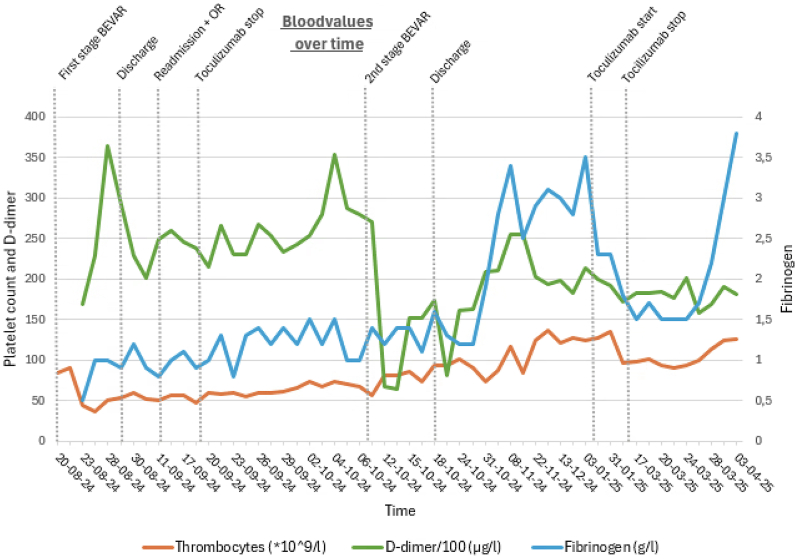
Blood values (thrombocytes, D-dimer/100, and fibrinogen) over time, with key clinical events indicated above. *BEVAR*, Branched endovascular aneurysm repair; *OR*, the surgery performed for the pseudo-aneurysm in her right groin.

Due to persistent hemoglobin (Hb) decline, thrombocytopenia, and remarkable large hematomas (Fig 2) on the third postoperative day, internal medicine was consulted. DIC was suspected and confirmed by a score of 5 using the International Society on Thrombosis and Haemostasis overt DIC scoring system (platelet count, 37 × 10^9^/L; D-dimer 16.883 μg/L; prothrombin time, 14.7 seconds; and fibrinogen 0.5 g/L). Platelets and fibrinogen were administered maintaining trough levels of platelets (>50 × 10^9^/L, in case of bleeding and >30 × 10^9^/L without bleeding) and fibrinogen (>1.5 g/L in case of bleeding and >1.0 g/L without bleeding).^3^ The performance of fibrinogen, platelet, and D-dimer levels is presented in Fig 3. A 4 Ts score was calculated to assess the likelihood of heparin-induced thrombocytopenia, yielding a low probability. Therefore, no additional tests were performed, and prophylactic low molecular weight heparin was continued. A computed tomography angiography revealed no active bleeding.

**Fig 4:**
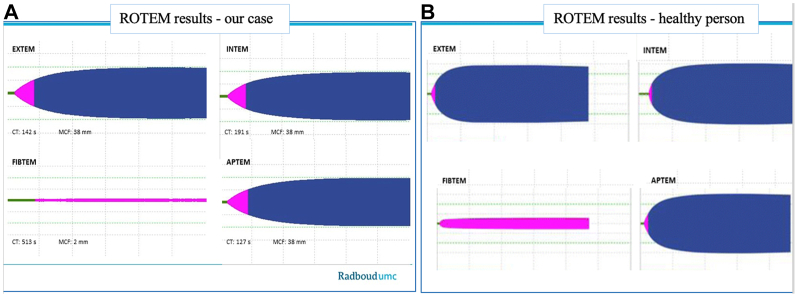
**(A)** The rotational thromboelastometry (*ROTEM*) results from our case during the surgical procedure for a pseudo-aneurysm in the right groin (11-9-24). Clotting time (*CT*) intervals are prolonged for all four tests, suggesting that the initiation of clotting is impaired. Maximum clot firmness (*MCF*) is abnormally low for EXTEM, INTEM, and APTEM, indicating a poor clot stability attributed to either low or dysfunctional platelets, low fibrinogen or hyperfibrinolysis. The FIBTEM is very low (2 mm), which suggests that in the absence of platelet function there would be virtually no clot formation, indicating lack of fibrinogen. **(B)** ROTEM results from a normal healthy person.^4^

Ten days after discharge, the patient was readmitted due to persistent pain in her right groin, with an evidently enlarged pseudoaneurysm on computed tomography angiography. A small arterial defect was primarily sutured, resolving the pseudoaneurysm. Platelet transfusion was administered prior to surgery. During surgery, a rotation thromboelastometry revealed low fibrinogen levels, confirmed by blood examination (Fig 4, *A* and *B*). With an additional low platelet count (84 × 10^9^/L), platelet transfusion and fibrinogen concentrate were given.

Additional diagnostic tests excluded hemolysis, thrombotic thrombocytopenic purpura, atypical hemolytic-uremic syndrome, myelodysplastic syndrome, and malignancy. Hemolysis was excluded based on normal lab results (Hb, 5.1 mmol/L; lactate dehydrogenase, 321 U/L; total bilirubin, 11 μmol/L; direct bilirubin, 4 μmol/L; haptoglobin, 0.45 g/L; reticulocytes 137.5 × 10^9^/L) and the absence of fragmentocytes. Normal ADAMTS13 activity (77%) excluded thrombotic thrombocytopenic purpura, and normal complement factor levels excluded atypical hemolytic-uremic syndrome. Bone marrow biopsy showed no relevant abnormalities, no M-protein was detected, and a positron emission tomography-CT excluded malignancy.

Tocilizumab was temporarily discontinued, as several previous cases report low fibrinogen levels during the use of tocilizumab. A high dose of prednisolone (40 mg) was administered to keep the GCA suppressed.^5^^,^^6^

**Fig 5:**
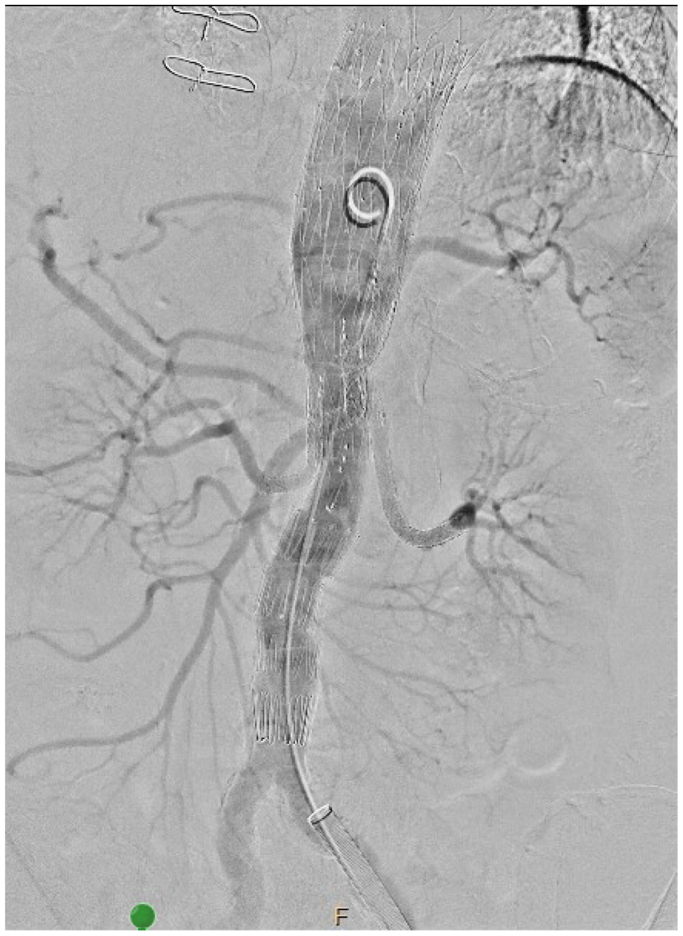
Completion angiography following the second stage of the branched endovascular aneurysm repair (BEVAR), demonstrating successful completion of the celiac trunk branch. No endoleak is observed.

After excluding most causes and considering the patient’s desire to treat her aneurysm, a multidisciplinary discussion concluded to perform the second stage. Two Begrafts Plus were placed to perfuse the celiac branch, with no endoleak on completion angiography (Fig 5). Over the next 2 weeks, her whole blood count remained stable, and no transfusions were required. The DIC gradually resolved weeks after the completion of the BEVAR and discontinuation of tocilizumab. A few months later, tocilizumab was reintroduced gradually to taper corticosteroid therapy, as the patient experienced steroid-related side effects. Fibrinogen levels decreased during therapy, after which we discontinued tocilizumab again. About 13 days after cessation, fibrinogen levels began to normalize (Fig 3).

## Discussion

Identification of the underlying cause of DIC is crucial for effective treatment. In this case, we propose a two-hit mechanism: chronic tocilizumab therapy likely led to subclinical hypofibrinogenemia, serving as the first hit, whereas the initial stage of the two-staged BEVAR, resulting in a type III endoleak and altered intra-aneurysmal blood flow, constituted the second hit.

Several cases of tocilizumab-induced hypofibrinogenemia have been reported,^6^ including one case involving treatment for GCA.^7^ Cytopenias are common side effects,^8^ whereas fibrinogen depletion is less well-known. Tocilizumab reduces platelet count by binding the interleukin-6 receptor, which normally stimulates thrombopoiesis.^9^ The mechanism behind fibrinogen reduction remains unclear. Immune-mediated hematologic effects may occur even after prolonged tocilizumab exposure. In our case, rechallenge resulted in a fibrinogen decline, which stabilized following discontinuation, supporting the hypothesis that tocilizumab contributed to the onset of DIC.

DIC following endovascular aortic repair (EVAR) is rare. Schizas et al reported 17 cases of DIC post-EVAR, with endoleaks as the primary cause in 71% of cases. Patients undergoing endovascular interventions for endoleaks responded well to treatment. The mortality rate was 29.4%, with no significant impact of treatment on survival.^10^ Other studies have reported that 2% to 4% of patients with abdominal aortic aneurysms develop DIC, often resolving with endovascular treatment.^11^

Several mechanisms have been proposed to explain the association between endovascular procedures and DIC. Dobrowolska et al suggested that change in blood flow within the aneurysm sac during EVAR triggers coagulation and fibrinolysis, leading to DIC.^12^ An endoleak is another potential cause, by creating altered, turbulent blood flow within the aortic sac, continuously activating the coagulation system.^10^^,^^13^ Additionally, exposure of blood to the denuded aortic endothelial surface has been observed in aneurysm-induced DIC.^14^ In our case, the aneurysm remained in circulation as the truncus branch was deliberately left open, causing a type III endoleak. Importantly, the prior ascending aortic repair had been performed as a single-stage procedure, during which no endoleak occurred.

In this case, a dual-track therapeutic approach was implemented. Tocilizumab was replaced with high-dose prednisone, and the BEVAR was completed, which also resolved the endoleak. Following these interventions, the DIC gradually resolved within several weeks.

## Conclusions

This case highlights the potential for DIC in patients undergoing EVAR while on long-term tocilizumab therapy. In patients receiving tocilizumab who develop signs of bleeding, fibrinogen levels should be evaluated. Perioperatively, maintaining fibrinogen >1.0 g/L (or >1.5 g/L in case of bleeding) is recommended. Temporary discontinuation of tocilizumab may be considered if fibrinogen is low and clinically acceptable. These measures may help prevent overt coagulopathy in high-risk patients.

## Funding

None.

## Disclosures

None.
